# Design of Novel Ceramic Preconcentrator and Integration in Gas Chromatographic System for Detection of Ethylene Gas from Ripening Bananas

**DOI:** 10.3390/s18082589

**Published:** 2018-08-07

**Authors:** Nayyer Abbas Zaidi, Muhammad Waseem Tahir, Micheal J. Vellekoop, Walter Lang

**Affiliations:** 1 International Graduate School for Dynamics in Logistics (IGS), University of Bremen, 28359 Bremen, Germany; wtahir@imsas.uni-bremen.de (M.W.T.); wlang@imsas.uni-bremen.de (W.L.); 2 Institute for Microsensors, -Actuators and -Systems (IMSAS), University of Bremen, 28334 Bremen, Germany; mvellekoop@imsas.uni-bremen.de

**Keywords:** ceramic preconcentrator, micro gas chromatographic system, ethylene gas

## Abstract

In this paper, a novel ceramic preconcentrator is manufactured using aluminum nitride (ALN) ceramics. The preconcentrator consists of a heater, a preconcentrator body, a gas inlet and a gas outlet. The adsorption material, Carbosieve SII, is loaded into the preconcentrator. The preconcentrator is integrated with a previously developed micro gas chromatographic system filled with ethylene. When operated, adequate ethylene gas is adsorbed into the preconcentrator. The application of heat pulse also successfully desorbs the ethylene gas. Tests are conducted with ethylene gas at concentrations of 10 ppm, 5 ppm and 2.5 ppm and 400 ppb, respectively. The system is also tested with ethylene gas from ripening bananas over a period of three days. No interference signal is observed in the chromatogram because of other ripening gases (e.g., carbon dioxide, oxygen, alcohol) and humidity. A detection limit of 25 ppb is realized with this system. The developed preconcentrator has several applications, e.g., in food industry and environmental monitoring.

## 1. Introduction

It is estimated that one-third of food, which is approximately 1.3 billion tons, is wasted annually in the world. Food losses, especially in fruits and vegetables in a food supply chain, are divided into five waste segments (i.e., food waste during agriculture, postharvest, processing, distribution and consumption). Fruits and vegetable losses during the transportation, handling and storage are known as postharvest losses, varying between 1% and 8% (depending upon the region) of the total loss [1,2,3].

Postharvest losses are caused by several factors, such as temperature, humidity, carbon dioxide, oxygen and excessive production of ethylene gas. Ethylene gas is a very influential factor in postharvest losses, and is emitted from fruits during the ripening of fruits and vegetables. Release of ethylene from over-ripped fruit accelerates the ripening of other fruits placed in the same enclosure, which means that in a cargo container, if several bunches of fruit start early ripening, they can initiate the ripening of nearby fruits, and thus, early ripening could damage the whole shipment, incurring significant losses. The release of ethylene, however, is in extremely low concentrations (parts per billion (ppb) levels); hence, it is always undetected until significant damage to fruit consignment is caused. It is reported that, in order to predict early ripening in a container, an analytical system is required for detecting ethylene gas with a concentration as low as 50 ppb [4,5].

In literature, several methods are used for the detection of ethylene gas, which include electrochemical sensors, non-dispersive infrared spectroscopy, metal oxide gas sensors and gas chromatography; see [3,6] for a review. An ethylene gas concentration of 20-ppm is detected using non-dispersive infrared spectroscopy by Biasio et al. [7]. Similarly, Krivec et al. used a metal oxide MOX gas sensor for detection of ethylene gas at a concentration of 200 ppm in the system [8]. Furthermore, field-effect transistor-based sensors show a sensitivity of 25 ppm for ethylene gas [9].

A fundamental approach to selective detection of particular gas in a mixture of gases is based on a gas chromatography system. The system consists of a gas chromatographic column, an injection system and a gas sensor. The gas mixture enters into the gas chromatographic column through an injection unit. The stationary phase inside the gas chromatographic column interacts with the gases and separates all gas species at different time intervals, as they pass through the column. The separated gases, as well as impurities, arrive at the sensor at different time intervals, and the gas sensor detects and displays in the form of concentration peaks (the chromatogram). These chromatograms are further analyzed, and the concentrations of gases are determined by calculating the peak of the chromatograms. The disadvantage of the gas chromatographic system is its large size and hence cannot be used for most on-site applications. The advances in both MEMS and 3D (three dimensional) printing technologies have enable scientists to fabricate a cheap gas chromatographic column, which significantly reduces the size of the gas chromatographic system [10,11].

Nasreddine et al. propose a portable gas chromatographic system for the detection of benzene, toluene, ethylbenzene and xylene (BTEX) [12,13]. A micro gas chromatographic system for the detection of ethylene gas presented by Janssen et al. suffered from baseline drift, which results in an inaccurate peak measurement of ethylene gas chromatogram at ppb concentration levels [5]. In our publication, we have shown the removal of baseline drift from ethylene gas chromatographic system [14]. Furthermore, metal oxide gas sensors in gas chromatographic systems show significant sensitivity towards humidity. A variation in humidity results in the change in the sensitivity of the metal oxide gas sensor. To reduce the effect of humidity, the metal oxide gas sensor is replaced by the electrochemical ethylene gas sensor; and the results of the micro gas chromatographic system become stable and are presented in [15]. 

**Figure 1:**
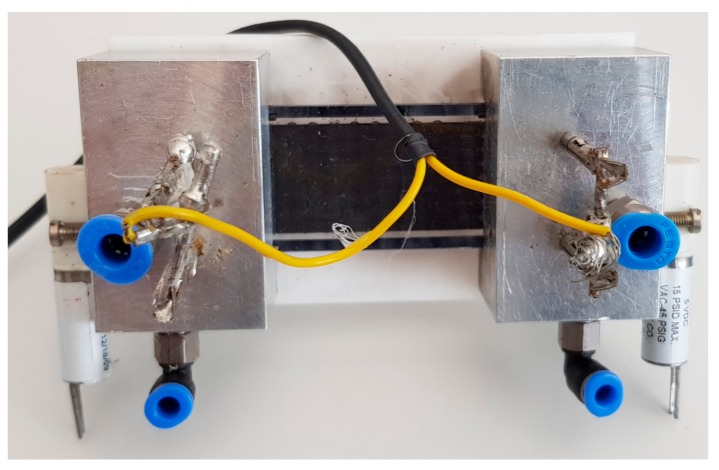
Silicon fabricated preconcentrator enclosed in housing [5].

For high sensitive detection of a particular gas in a gas chromatographic system, the addition of a preconcentrator is essential. A preconcentrator not only removes the injection system from the chromatographic system, but also makes a system more sensitive to gas detection. The preconcentrators adsorb a certain gas for a period of time, application of heat pulse by means of its heater results in the desorption of the adsorbed gas. The advantages of preconcentrators are their simple attachment to gas chromatographic systems. Conventional preconcentrators are glass tubes filled with adsorption materials and wires wrapped around the tubes to provide heat. The MEMS technology minimizes the use of the large tube and makes it possible to fabricate custom design preconcentrators with an attached heater on the backside of a small-size chip. The work in the use of preconcentrator for ethylene detection at the ppb concentration scale was presented in 2012 by Dow et al. [16]. The preconcentrator is fabricated using silicon technology. The preconcentrator is made by two-layer glass silicon bonding with a platinum heater on the bottom. In 2014, a large-capacity on-chip preconcentrator to concentrate ethylene gas was presented by Janssen et al.; see [5] for details of the preconcentrator design. It requires housing, which consists of a Teflon plate and two aluminum parts, as shown in Figure 1. The two aluminum parts have two inlets, two outlets and two solenoid valves. Their combination directs the flow towards the preconcentrator or makes the flow bypass the preconcentrator.

The use of the silicon fabricated preconcentrator shows results with drifts for the detection of ethylene gas; see [5] for previous work on the preconcentrator. The production of miniaturized preconcentrator by silicon technology is difficult and expensive. However, two problems are associated with the silicon fabricated preconcentrator.

1. Firstly, heating the preconcentrator heats the housing that generates unnecessary drifts in the ethylene chromatogram; see [5] for details of drifts in a chromatogram using the silicon preconcentrator.
2. Secondly, at the level of research and development, even a faster and simpler solution is always an area of thrust. The disadvantage linked with the silicon technology is higher costs in running the system.

In this paper, authors present a preconcentrator design, and its fabrication. Its thermal and chemical characteristics are tested before it is integrated in a micro gas chromatographic system for the detection of ethylene gas. The paper is structured as follows. Section 2 briefly explains ceramic technology. Section 3 describes the design of the ceramic preconcentrator. Section 4 briefly explains thermal characterization of the ceramic preconcentrator. Section 5 discusses the chemical characterization by integrating the preconcentrator into the gas chromatographic system. Section 6 elaborates the results obtained from the gas chromatographic system. Finally, Section 7 explains the conclusion and future work.

## 2. Ceramic Technology

The ceramics technology offers a wide range of applications in automotive industry, electronics, energy and environmental technology, mechanical and medical engineering. In automotive industry, piezo-ceramic sensors in vehicles provide information about vehicle engines, positions and changes in direction. In electronics, ceramic heat sinks deliver right environment for power electronics components. In environmental technology, ceramic materials have outstanding resistance against corrosion; their properties related to temperature and wear have several applications in power plants, wind turbines and photovoltaic. In medical engineering, ceramic materials with biocompatible and wear-resistant features offer applications in hips, shoulder joints, etc. [17,18,19,20].

The new developments in the area of ceramic technology open the realization of new possibilities for preconcentrator design. The manufacturing costs and the production time are lesser by means of this technology. However, the challenges are shown in the following aspects: (1) the stability of a preconcentrator with a temperature range between 150 °C and 200 °C; (2) adequateness of adsorption and desorption of ethylene gas; (3) gas leakage resistance of the gas inlet and outlet of the preconcentrator; and (4) generation of an interfering signal on the sensor, when the preconcentrator is heated and a constant speed of the carrier gas between 10 standard cubic centimeters per minute (sccm) and 20 sccm is applied to the preconcentrator with a pressure of 1.3 bars. The danger with ceramic preconcentrators is that they outgas, in particular by the heating, and thus, the measuring signal is inaccurate or an additional noise signal is generated.

## 3. Ceramic Preconcentrator Design

To select a ceramic material for the preconcentrator, a few essential aspects must be kept in mind. The material needs to exhibit good thermal conductivity, so that sufficient heat pulses desorb the gas from the preconcentrator. It must be electrically insulated; the printed heater on the surface of the ceramic preconcentrator must not short-circuit upon running of the power through it. The ceramic technology at CeramTec offers several materials for the fabrication of ceramic chips, which include aluminum oxide, aluminum titanate, aluminum nitride; see [17] for material selection. The ceramic technology that fulfills the preconcentrator criteria is aluminum nitride with a metal layer of tungsten. Aluminum nitride has a thermal conductivity of 200 W/mK with high electrical insulation (1.10 TΩ·cm) and the metal layer has a sheet resistance of 95 mΩ/square.

**Figure 2:**
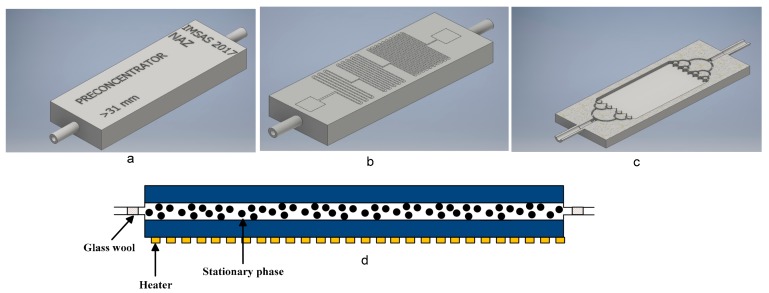
(**a**) Frontside; (**b**) backside; (**c**) sliced view; (**d**) side and sliced view of the preconcentrator filled with an adsorption material.

For the design of the preconcentrator, it is paramount to design a single unit that can be easily integrated into a micro gas chromatographic system. A preconcentrator was designed with CAD (computer Aided Design) software. The designed preconcentrator was rectangular cuboid; its frontside and backside are shown in Figure 2a–c. The backside of the preconcentrator contained a heater, which was designed in three sections for homogeneous heat distribution. Two pads were used for electrical connection, and two hoses were used for the gas inlet and outlet. Inside the preconcentrator was an empty rectangular volume that was filled with an adsorption material. The dimensions of the preconcentrator are shown in Table 1.

There are several adsorption materials which are available from several manufacturers. Carbosieve SII gives good results when tested for the adsorption of ethylene gas [21]. The adsorption material was loaded into the preconcentrator using a vacuum pump. To confine the adsorption within the preconcentrator, superfine glass wool (Glasswarenfabrik, Karl Hecht GmbH & Co KG) was used at both ends, as shown in Figure 2d. The amount of Carbosieve SII filled in the preconcentrator was 1.187 g.

**Figure 3:**
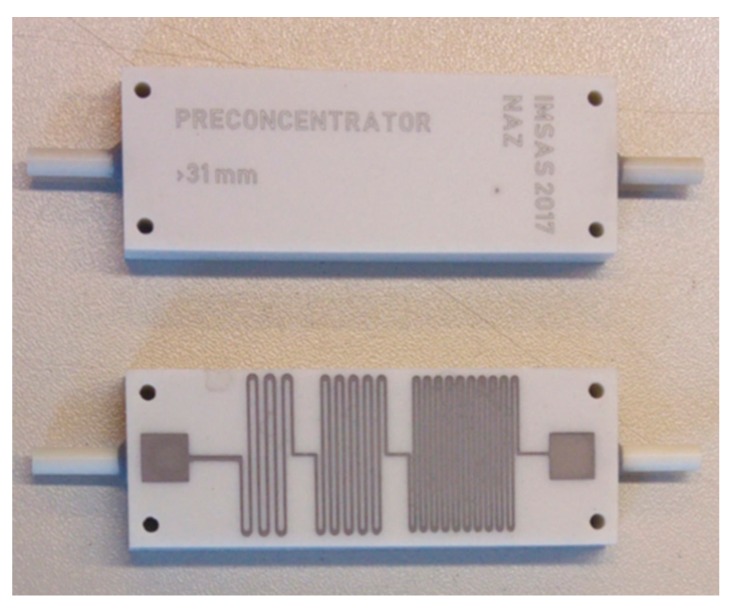
Frontside and backside of the ceramic preconcentrator.

The chip was manufactured using ceramic technology at Ceramtec, as shown in Figure 3. It consisted of two pieces, which formed a single unit by co-firing. The two hoses were glued to the two ends of the device. A metallic heater screen printed on the backside of the ceramic preconcentrator chip was divided into three sections with a total resistance of 132 Ω ± 4 Ω.

## 4. Thermal Characterization

**Figure 4:**
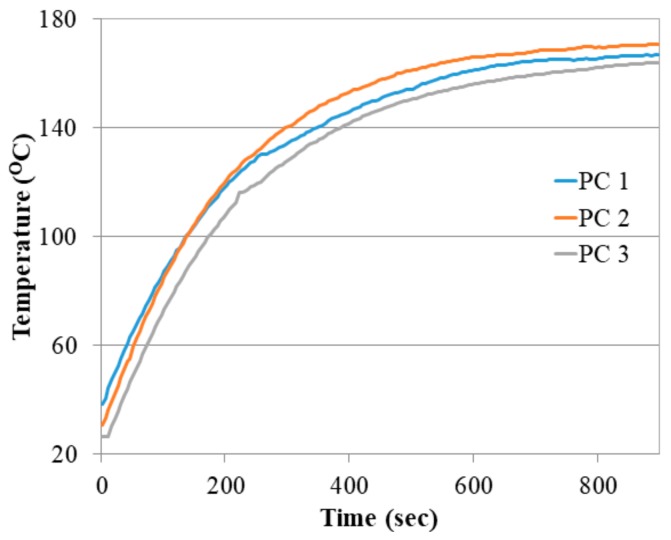
Temperature as a function of time for three different preconcentrator chips applied with a maximum consuming power of 18 W and a voltage of 50 V.

**Figure 5:**
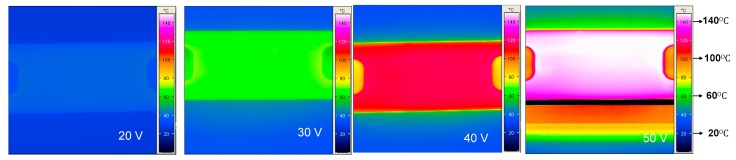
Thermal characterization of the preconcentrator heater at 20 V, 30 V, 40 V and 50 V, respectively.

The preconcentrator was placed under a thermal imaging camera (VarioTHERM, infraTec, Dresden, Germany) to study the thermal characteristics of the ceramic preconcentrator heater. Figure 4 shows the temperature change of three preconcentrator chips when a maximum power of 18 W was applied. It was observed that the maximum temperature of 150 °C at a voltage of 50 V was achieved in approximately 600 s. Furthermore, in order to see the temperature distribution of the preconcentrator, a range of voltage (20–50 V) was applied in steps of 10 V until the temperature reached 150 °C with a holding time of 10 min, as shown in Figure 5.

## 5. Chemical Characterization

**Figure 6:**
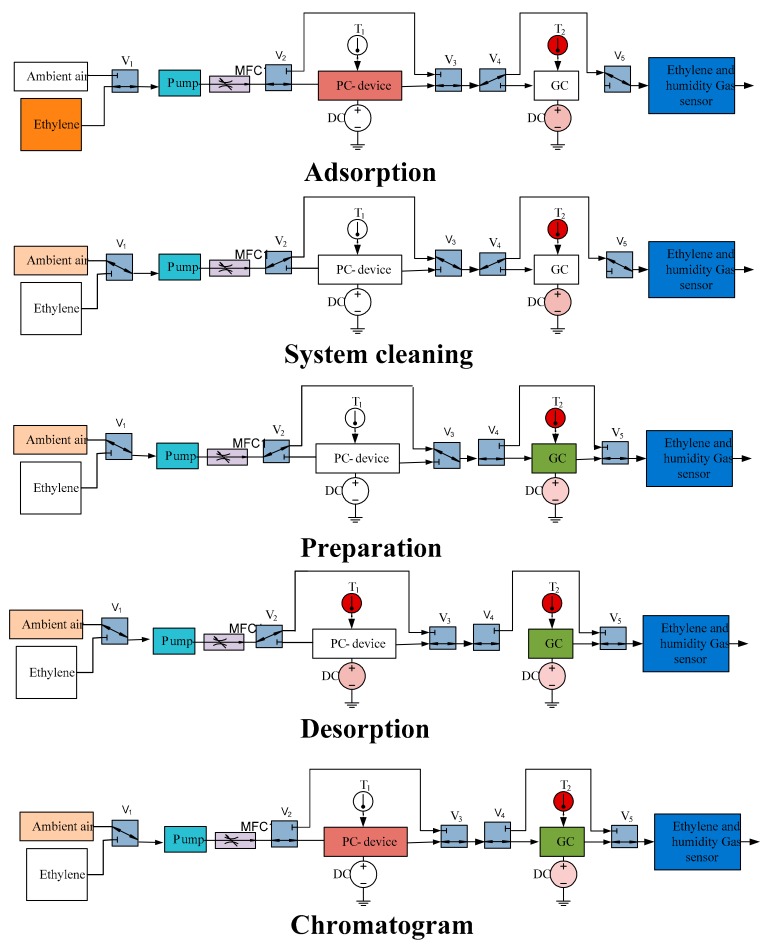
Gas chromatographic system operation. From top to bottom: adsorption, system cleaning, preparation, desorption and chromatographic step.

The ceramic preconcentrator was integrated into the ethylene gas chromatographic system for its chemical characterization. The block diagram of the system is shown in Figure 6. The system consisted of five valves (V1 to V5), a micro gas chromatographic column filled with Carbosieve SII as a stationary phase, a pump, a mass flow controller, an electrochemical ethylene gas sensor and a humidity sensor. The valves were used to switch between the gases. The gases used in the system were ethylene gas and ambient air. The pump was used to draw air or ethylene from the environment and send it to the chromatographic system. The flow rate of the gases was controlled at 10 sccm by the mass flow controller. 

The gas chromatographic system worked in the following steps, and Figure 6 shows each step according to the ‘pass’ and ‘bypass’ positions of the valve.

(1) **Adsorption:** initially, the ethylene gas was connected to the system and the gas chromatographic column was bypassed. The ethylene gas entered into the preconcentrator, bypassed by the gas chromatographic column, and entered into the electrochemical gas sensor. It took 1200 s for ethylene to be adsorbed in the preconcentrator.
(2) **Cleaning:** the preconcentrator was bypassed and the ambient air was connected. This step cleaned the remaining ethylene in the tubes. This step took around 600 s.
(3) **Preparation:** the gas chromatographic column was connected. In this step, the preconcentrator was bypassed and the ambient air was connected. The baseline of the system was obtained. This step took around 600 s.
(4) **Desorption:** the preconcentrator was heated to 150 °C for 600 s. Heating the preconcentrator resulted in the desorption of ethylene gas.
(5) **Chromatogram:** the preconcentrator was connected. The ambient air took the ethylene gas from the preconcentrator to the gas chromatographic column. The preconcentrator was connected with the chromatogram system for 180 s before disconnected with the system.

**Figure 7:**
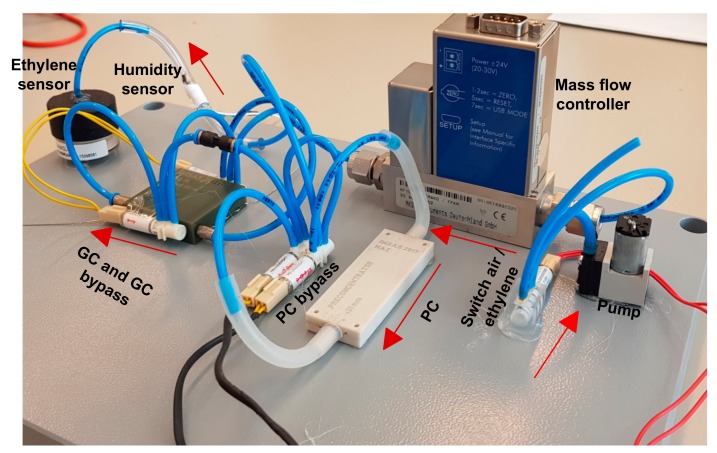
Gas chromatographic system.

The individual components used in the gas chromatographic system are following: the material used in the gas chromatographic column and preconcentrator was Carbosieve SII with a mesh size of 80–100 nm. It was purchased from Sigma-Aldrich [21]. The ethylene cylinder with a concentration of 400 ppb was purchased from Air Liquide [22]. The valves used in the system were purchased from The LEE Company [23]. The pump with a flow rate of 500 sccm [24] was used in the system and was bought from Schwartz Precison Manufacturing. This flow rate was reduced to 10 sccm by using a mini mass flow controller purchased from MKS Instrument, Inc [25]. The gas chromatographic column used in the system was printed using a 3D printer. Plastic was used as a printing material for the gas chromatographic column printing (see column details in [10]). The ceramic preconcentrator was manufactured from Ceramtec [17]. The electrochemical ethylene gas sensor and the humidity sensor were from Membrapor and Sensirion, respectively [25,26]. The system is shown in Figure 7.

## 6. Results and Discussion

**Figure 8:**
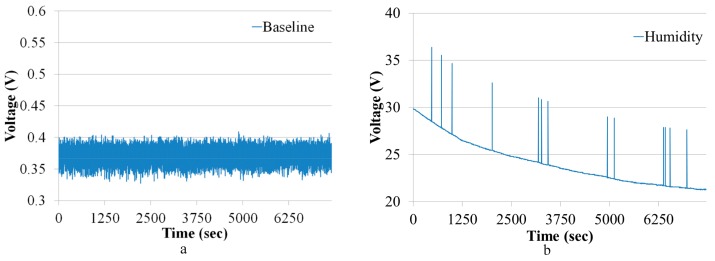
(**a**) Baseline of the gas chromatographic system; (**b**) baseline from the humidity sensor.

Figure 8a shows the baseline of the gas chromatographic system obtained using ambient air as carrier gas. The gas chromatographic column was connected, the preconcentrator was bypassed, and the baseline of 7400 s was obtained. The flow rate was fixed at 10 sccm. The data from the humidity sensor during the baseline measurement are shown in Figure 8b. There was a drift in the baseline of the humidity sensor. This baseline drift could be a result of humidity adsorbed in the gas chromatographic column and the variation in the humidity of ambient air. However, the drift in the humidity has no effect on the baseline from the ethylene gas sensor, as claimed earlier in the introduction section. 

**Figure 9:**
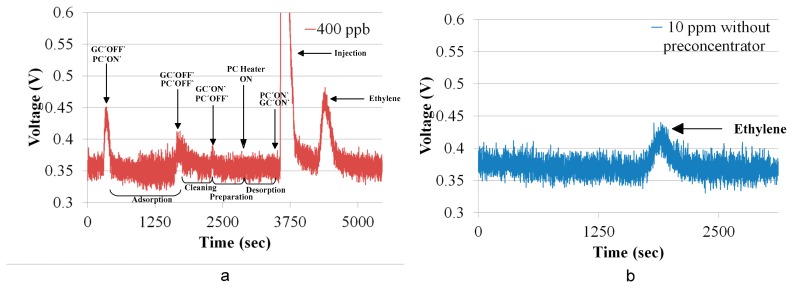
(**a**) Chromatogram of ethylene at 400 ppb; (**b**) chromatogram of ethylene at 10 ppm without the preconcentrator.

**Figure 10:**
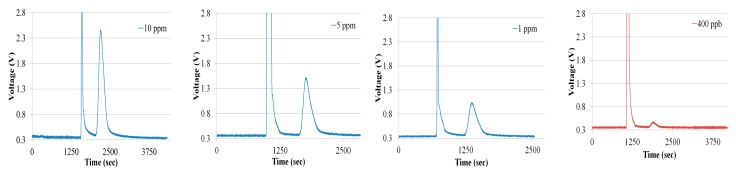
Gas chromatographic system characterization at 10 ppm, 5 ppm, 1 ppm and 400 ppb, respectively.

Figure 9a shows the chromatogram of ethylene at a concentration of 400 ppb. Adsorption of ethylene gas was carried out for 1200 s, and the times for system cleaning, preparation and desorption were all 600 s. The initial peaks were attributable to the switching between the preconcentrator and the gas chromatographic column, as the switching altered the flow rate in the gas chromatographic system. The injection peak was obtained after the desorption and the peak next to injection peak was the ethylene peak. The total time taken for a single measurement was 5000 s. To investigate the effectiveness of the preconcentrator, ethylene with a concentration of 10 ppm and a volume of 2.7 mL was injected into the gas chromatographic system without the preconcentrator. The chromatogram of ethylene with a concentration of 10 ppm is shown in Figure 9b. The peak of chromatogram of ethylene at 400 ppb using a system with the preconcentrator was higher than that of ethylene chromatogram with a concentration of 10 ppm. The system was further tested at higher concentrations of 10 ppm, 5 ppm, 1 ppm and 400 ppb. The ethylene chromatograms are shown in Figure 10. The ethylene gas cylinders with 10 ppm and 400 ppb are commercially available [22]. To prepare ethylene gas with concentrations of 5 ppm and 1 ppm, we mixed 10 ppm ethylene gas with synthetic air. For example, 5 ppm ethylene is prepared by mixing 0.5 L ethylene with 0.5 L synthetic air in a gas bag [15].

**Figure 11:**
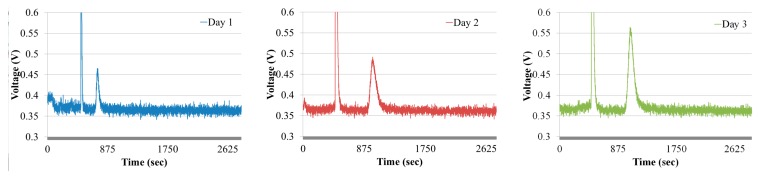
Ethylene chromatograms from ripening bananas.

To evaluate the effectiveness of the gas chromatographic system in the presence of interfering gases, ethylene gas was extracted from ripening bananas. Bananas with a weight of 0.2 kg were stored in a sealed box with a volume of 50 L. Each day, ethylene gas was extracted from the box for a period of three days. The chromatograms obtained from the ripening bananas are shown in Figure 11. It is evident that no interference signal was generated because of the interference gases, like carbon dioxide and oxygen, and humidity, as seen in the chromatograms.

**Figure 12:**
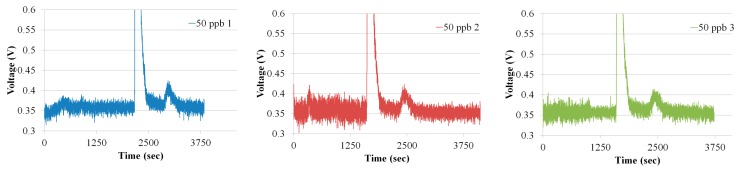
Three ethylene chromatograms at 50 ppb.

To further analyze the system repeatability and the resolution, the efficiency of the preconcentrator was increased by increasing the flow rate to 20 sccm during adsorption. Increasing the flow rate resulted in an increase in the adsorption of ethylene in the preconcentrator. The ethylene concentration of 50 ppb was prepared by mixing 400 ppb ethylene gas with synthetic air; see [15] for details of the mixing procedure. Figure 12 shows the three chromatograms of ethylene at 50 ppb. The statistical calculation revealed that a minimum concentration of 25 ppb was detected though this system and the system showed good repeatability.

## 7. Conclusions and Future Work

A single-unit ceramic preconcentrator was manufactured. The system showed no interference signal during adsorption and desorption. No gas leakage was observed. The thermal and chemical characterizations of the preconcentrator were presented. The preconcentrator was integrated into the micro gas chromatographic system, which consisted of a micro gas chromatographic column filled with Carbosieve SII as a stationary phase, five valves, a pump, a humidity sensor, an electrochemical ethylene gas sensor and a mini mass flow controller. A detection limit of 25 ppb was realized with this system. The developed portable system has many benefits, including cutting food waste and reducing emission of harmful gases (greenhouse gases causing global worming) into the environment during decaying of the fruits and vegetables, and hence can find many applications in fields including food industry. 

In future work, the system will be studied with ripening bananas stored in a box for a period of three weeks.

## Figures and Tables

**Table 1:** Dimensions of the ceramic preconcentrator.

Inner Diameter of Hose	2 mm
Outer Diameter of Hose	3.5 mm
Length of Hose	10 mm
Resistance of Heater	143.8 Ω
Size of Chip	61 mm × 23 mm × 5.5 mm
Diameter of Channels	0.5 mm
Heater Thickness	14 um
Heater Length 1	388 mm
Heater Width 1	0.32 mm
Heater Length 2	194 mm
Heater Width 2	0.32 mm
Heater Length 3	97 mm
Pad Size	6 mm × 6 mm
Width of Channel	0.5 mm
